# Biosynthesis Microwave-Assisted of Zinc Oxide Nanoparticles with *Ziziphus jujuba* Leaves Extract: Characterization and Photocatalytic Application

**DOI:** 10.3390/nano11071682

**Published:** 2021-06-26

**Authors:** Maymounah N. Alharthi, Iqbal Ismail, Stefano Bellucci, Nezar H. Khdary, Mohamed Abdel Salam

**Affiliations:** 1 Department of Chemistry, Faculty of Science, King Abdulaziz University, P.O. Box 80200, Jeddah 21589, Saudi Arabia; chem-28@hotmail.com (M.N.A.); iismail@kau.edu.sa (I.I.); 2 Department of Chemistry, College of Science, Princess Nourah bint Abdulrahman University, P.O. Box 84428, Riyadh 11671, Saudi Arabia; 3 National Laboratories of Frascati, National Institute of Nuclear Physics, I-00044 Frascati, Italy; stefano.bellucci@lnf.infn.it; 4 King Abdulaziz City for Science and Technology, P.O. Box 6086, Riyadh 11442, Saudi Arabia; nkhdary@kacst.edu.sa

**Keywords:** Azo dye, photocatalytic degradation, solar irradiation, ZnO NPs

## Abstract

The present work is intended to biosynthesize zinc oxide nanoparticles (ZnO NPs) via facile and modern route using aqueous *Ziziphus jujuba* leaves extract assisted by microwave and explore their photocatalytic degradation of methyl orange anionic dye and methylene blue cationic dye under solar irradiation. The biosynthesized microwave assisted ZnO NPs were characterized and the results showed that ZnO NPs contain hexagonal wurtzite and characterized with a well-defined spherical-like shape with an outstanding band gap (2.70 eV), average particle size of 25 nm and specific surface area of 11.4 m^2^/g. The photocatalytic degradation of the MO and MB dyes by biosynthesized ZnO NPs under solar irradiation was studied and the results revealed the selective nature of the ZnO NPs for the adsorption and further photocatalytic degradation of the MO dye compared to the MB dye. In addition, the photocatalytic degradation of MO and MB dyes by the ZnO NPs under solar radiation was fitted by the first-order kinetics. Moreover, the photodegradation mechanism proposed that superoxide ions and hydroxyl radicals are the main reactive species.

## 1. Introduction

Currently, nanotechnology is based on the preparation, characterization and applications of different nanoparticles (NPs) such as metals, metal oxides, semiconductors, ceramics and polymers, due to their outstanding morphological, structural and physicochemical properties allowed the NPs to be the used in a wide variety of applications. Among the most commonly used nanoparticles is zinc oxide nanoparticles (ZnO NPs), which are usually used in different applications, such as corrosion protection [1], food packaging [2], biomedical [3,4,5], electronics [6], revolutionizing agriculture [7], textiles [8] and as photocatalysts for environmental applications [9,10,11,12,13]. The synthesis of ZnO NPs are usually based on various physical, and chemical process, such as via combustion, thermal decomposition, sol-gel method, mechanical synthesis combined with high-energy milling and hydrothermal methods, which are mainly based on the usage of different chemicals and reagents such as the aqueous solution of zinc nitrate, zinc sulphate or zinc acetate as the precursor, followed by provision of basic environment such as sodium hydroxide or ammonia, in addition to some additives acting similarly to capping agents to produce well defined nanoparticles such as polyethylenimine (PEI) or polyethylene glycol (PEG) [14,15,16]. The physical and chemical processes for synthesis of ZnO NPs usually generate chemical waste, leading to adverse effects on the life of flora and fauna and, critically, water. Recently, an alternative and interesting trend was adopted to minimize the generation of such chemical waste known as biosynthesis, or green synthesis, of nanoparticles, where various plant parts extract have been used for this synthesis, as the biosynthesis of ZnO NPs in comparison with conventional synthesis is recommended, as it has minimal impact on the environment and is without health risk [17]. In several studies, the extract of various plants parts have been applied for the biosynthesis of ZnO NPs, such as *Azadirachta indica* (Neem) leaf [18], *Abelmoschus esculentus* (okra) *mucilage* [19], *Cuminum cyminum* (cumin) [20], *Mangifera indica* (mango) leaves [21], *Calotropis gigantea* leaves [22], *Aloe socotrina* leaf [23], *Parkia roxburghii* seeds [24], *Nigella sativa* seed [25] and *Ziziphus jujube* (Sidr or Nabq) [26], where the plant extracts act as capping and stabilizing agents [17], which stabilized the formed nanoparticles, and prevent the agglomeration of the particles. In addition, the biosynthesized ZnO NPs were characterized with comparable, and most of the times higher activities when compared with traditionally prepared nanoparticles [17,18,19,20,21,22,23,24,25,26,27]. *Ziziphus jujube* is a very common plant that is grown locally in Saudi Arabia and surrounding areas and is used extensively for its health properties [28,29,30,31]. It was found that the medicinal benefits of *Ziziphus jujube* are anti-inflammatory effects, antimicrobial, antioxidant and anticancer [28,29,30,31]. The *Ziziphus jujube* plant is rich in several organic compounds, including phenolic compounds, beta-carotene, alpha-tocopherol, alkaloids, sterols, flavonoids, saponin, tannins and fatty acids which could act as capping agent, and prevent the agglomeration of the NPs due to the existence of long chain natural products in the plant extract [32,33].

Moreover; the preparation of metal oxide NPs such as ZnO using microwave was the focus of many research, mainly due to the superior heating rate when compared to traditional heating methods as a result of volumetric heating, in addition to the fact that microwaves usually provide high energy by penetrating the material, allowing the reaction to be completed in minutes or even seconds, in addition to the possibility to control the ZnO NPs properties; such as purity, low cost, reproducibility of the produced NPs and fulfilment of the eco-friendly approach criterion as well. [34,35,36,37,38,39].

The hypothesis of the current work in the use of *Ziziphus jujube* leaves extract assisted with microwave could produce ZnO NPs characterized with outstanding colloidal stability, more uniform size, shape and higher reactivity compared to the traditionally prepared ZnO NPs. 

Accordingly, the objective of the current research is the biosynthesis of ZnO NPs using aqueous extract of *Ziziphus jujube* leaves assisted with microwave heating. The biosynthesized ZnO NPs were characterized using different characterization techniques to explore their morphological, physical and chemical properties, then used for the photocatalytically degradation of two different organic dyes: methyl orange anionic dye and the methylene blue cationic dye, under solar irradiation.

## 2. Materials and Methods

### 2.1. Materials

All chemicals (analytical grade) were purchased from Sigma-Aldrich Canada (Oakville, ON, Canada), and the solutions were prepared with deionized water.

### 2.2. Methods

#### 2.2.1. *Ziziphus jujuba* Leaves Extract (Sidr) Preparation

Fresh and healthy leaves of *Ziziphus jujuba* (Sidr) were collected from Jeddah, Saudi Arabia, on October 2019. The leaves were washed very well with tap water, then with deionized water and were dried on air at ambient temperature, then the cleaned leaves were chopped and grounded and 5.0 g was added to a 100 mL of deionized water in a beaker. The mixture was boiled for 20.0 min, then cooled to room temperature and was centrifuged at 3,600 rpm for 30 min, till a clear filtrate was obtained and stored at 4 °C.

#### 2.2.2. Traditional ZnO NPs (T ZnO NPs) Preparation

An aqueous solution of zinc acetate (250 mL, 0.2 M) and the solution of NaOH (250 mL, 0.5 M) were prepared with deionized water. The NaOH solution was added drop by drop using a burette to the zinc acetate solution at room temperature under vigorous stirring, which resulted in the formation of the white precipitate of zinc hydroxide. The white precipitate of the zinc hydroxide was separated by centrifugation at 3900 rpm for 30 min and washed three times with distilled water, followed by ethanol. The obtained product was dried at 60 °C in air atmosphere for 24 h to convert the Zn(OH)_2_ to ZnO NPs.

#### 2.2.3. Biosynthesis of ZnO NPs (B ZnO NPs)

An aqueous solution of Zinc acetate (250 mL, 0.2 M) and the solution (250 mL, 0.5 M) of NaOH were prepared using the *Ziziphus jujuba* leaves extract (5 mL). The NaOH solution was added drop by drop using a burette to the zinc acetate solution; prepared with the extract, at room temperature under vigorous stirring, which resulted in the formation of the light-brown precipitate of zinc hydroxide. The light-brown precipitate was separated by centrifugation at 3900 rpm for 30 min and washed three times with distilled water, followed by ethanol. The obtained product was dried at 60 °C in air atmosphere for 24 h to convert the Zn(OH)_2_ to ZnO NPs.

#### 2.2.4. Biosynthesis Microwave-Assisted ZnO NPs (BMW ZnO NPs)

Aqueous solution of zinc acetate (250 mL, 0.2 M) and the solution (250 mL, 0.5 M) of NaOH were prepared using the *Ziziphus jujuba* leaves extract (5 mL). The NaOH solution was added drop by drop using a burette to the zinc acetate solution; prepared with the extract, at room temperature under vigorous stirring at 1000 rpm using Stuart™ hotplate stirrer model CB162, which resulted in the formation of the light-brown precipitate of zinc hydroxide. The resultant solution was treated by using 800 W household digital microwave oven (Nikai, NMO-518N, Japan) for 5 min to convert the Zn(OH)_2_ to ZnO NPs, then the precipitate was separated by centrifugation using Sigma Centrifuge (model 2-7) at 3900 rpm for 30 min and washed three times with distilled water, followed by ethanol. The obtained product was dried at 60 °C in air atmosphere for 24 h.

### 2.3. Characterization Methods

The FT-IR spectra for the FT-IR spectra of *Ziziphus jujuba* leaves extract, and different ZnO NPs, were recorded using FTIR spectrophotometer (Shelton, CT, USA). X-ray diffraction (XRD) patterns were recorded for phase analysis and the measurement of crystallite size was performed on a Philips X-pert pro diffractometer (Malvern, Worcestershire, United Kingdom). The instrument was operated at 40 mA and at 40 kV on a CuK α radiation and a nickel filter in the 2θ range from 2 to 80° in steps of 0.02°, with a sampling time of one second per step. Estimation of the crystal size was achieved according to the Scherrer equation [40]. The morphological structures of the prepared ZnO NPs were studied using JEOL JEM-1011 high-resolution transmission electron microscope (TEM) (Peabody, MA, USA) that operated 80 kV. The specific surface area of the ZnO NPs were estimated using the nitrogen adsorption/desorption isotherm at 77 K, by NOVA3200e (Quantachrome, Boynton Beach, FL, USA), and prior to measurements, the samples were outgassed under vacuum (5 millitorrs) at were degassed at 473 K; the outgas rate was 5 mmHg/min till the sample was degassed. The absorbance and the band gap estimation were measured using a UV-3600 from Shimadzu (Nakagyo-ku, Kyoto, Japan). For absorbance measurement, 20 mg of the sample was mixed with 100 mg of KBr and then ground very well to prepare a transparent pellet. After that, the pellet was used for the measurement of absorbance.

### 2.4. Photocatalytic Experiments

The application of ZnO NPs as a photocatalyst for environmental remediation via photocatalytic degradation was performed using a mixture of the two azo dyes solution—methyl orange (MO) and methylene blue (MB), under direct sunlight. A 20 mL mixture of 5 ppm of MO and 5 ppm MB dyes were used, and 15 mg of ZnO NPs were added and stirred for 30 min in the dark, then the solution was exposed to direct sunlight between 10 am to 12 pm, and the light flux was 700 ± 20 W/m^2^ during the whole period. An aliquot of the mixture was withdrawn at designated time intervals, the ZnO NPs were separated by centrifuge and then the concentration of the remaining MO and MB dyes in each solution was determined by ultraviolet-visible spectrophotometer (UV-1650 PC, CPS-240A, SHIMADZU, Nakagyo-ku, Kyoto, Japan) at 464 nmand 664 nm, respectively. The removal efficiency was estimated by applying the following equation:

(1) $$\%\;\mathrm{Removal}\;=\frac{100\times \left(C_{0}-{\;C}_{t}\right)}{C_{0}}$$

where *C*_0_ is the initial concentration and *C_t_*: the residual concentration in solution at a certain time (*t*).

## 3. Results and Discussion

### 3.1. Characterization of ZnO NPs

**Figure 1:**
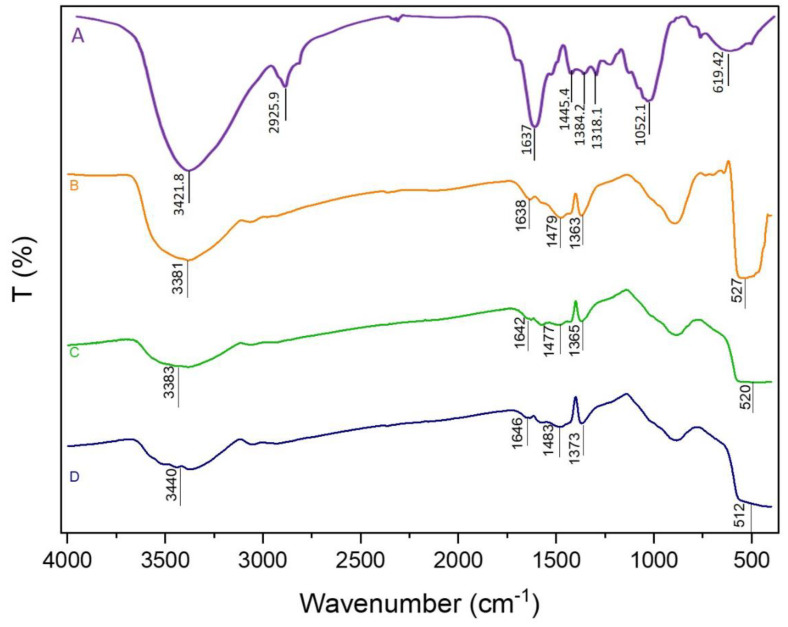
FT-IR spectra of Ziziphus jujuba leaves extract (**A**), T ZnO NPs (**B**), B ZnO NPs (**C**) and BMW ZnO NPs (**D**).

Figure 1 present the FT-IR spectra of *Ziziphus jujuba* leaves extract, as well as the different synthesized ZnO NPs. The spectrum of *Ziziphus jujuba* leaves extract (Figure 1A, showed strong absorption peak at 3421 cm^−1^ was resulted from stretching of the O-H groups due to the presence of alcohols, phenols, carbohydrates and etc. [41]. While the peak 2925 and 1637 cm^−1^ may be attributed to the stretching vibration of υ (=C–H) and υ(C = C), and the peak at 1637 cm^−1^ could also be related to the surface adsorbed water molecule. The peak at 619 cm^−1^ may be assigned to δ(C–H) bending vibration or C–S, R–C–CH_3_ stretching for Sulphur compounds. Similarly, the bands at 3421 and 1384 cm^−1^ may be assigned to the stretching vibration of υ (O–H) and in-plane bending vibration of δ (O–H), respectively. Moreover, the band at 1052 cm^−1^ may be contributed by skeletal C–O and C–C vibration bands of glycosidic and pyrenoid ring [42]. Meanwhile, the FT-IR spectra of different ZnO NPs were analyzed to confirm the phase transformation and purity of the ZnO (Figure 1B–D). All the FT-IR spectra showed a wide band at 3400 cm^−1^ thereby indicating the presence of surface hydroxyl groups due to presence of trace amount of water in the ZnO samples. Weak absorption bands centered at about 1640, 1480 and 1370 which can be assigned to asymmetric and symmetric C=O stretching modes, respectively, due to traces of the *Ziziphus jujuba* leaves extract, especially with the biosynthesized ZnO Nps. The peaks appeared in the region between 600 and 450 cm^−1^ are allotted to metal oxygen vibration (Zn-O) of ZnO nanoparticles [43].

**Figure 2:**
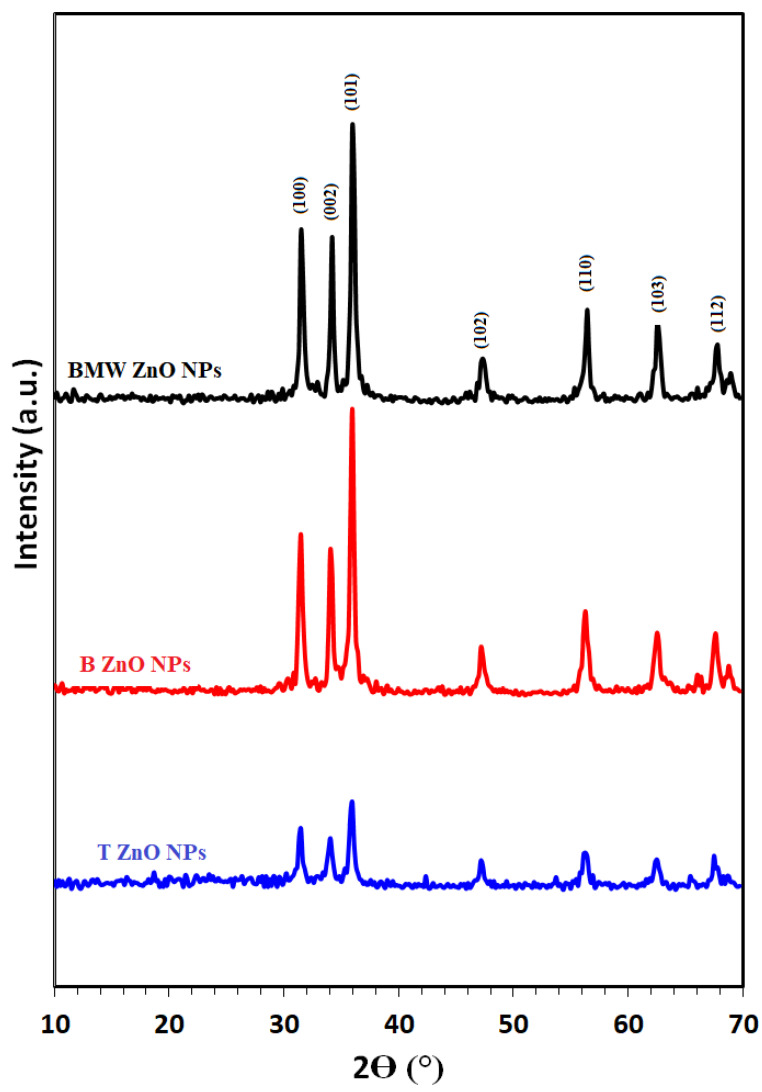
XRD patterns of different ZnO NPs.

**Figure 3:**
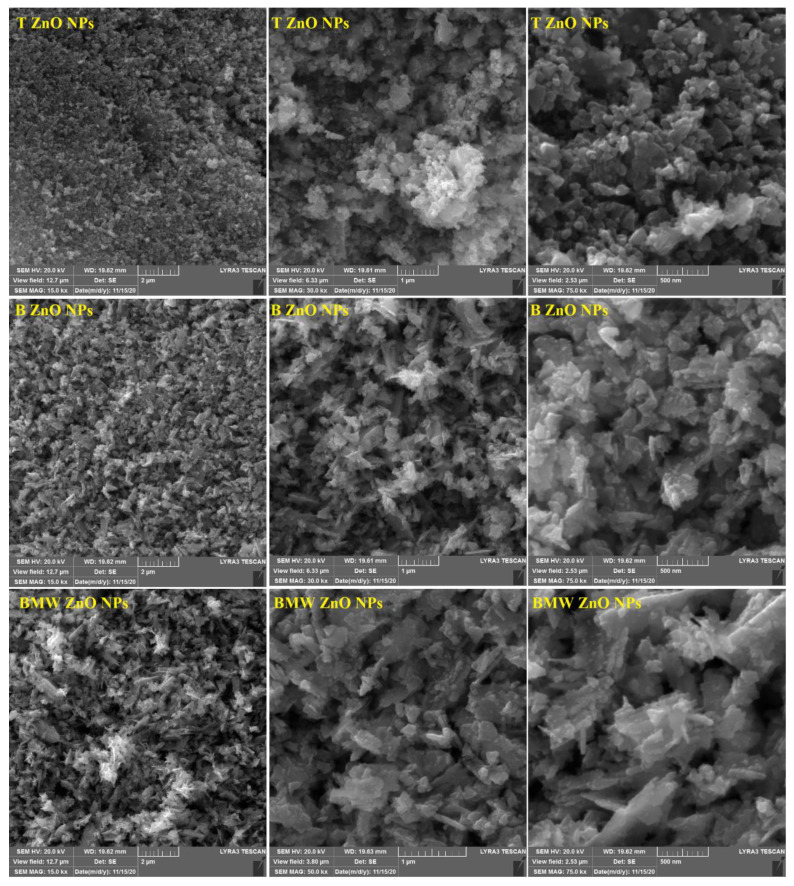
SEM micrographs of different ZnO NPs.

**Figure 4:**
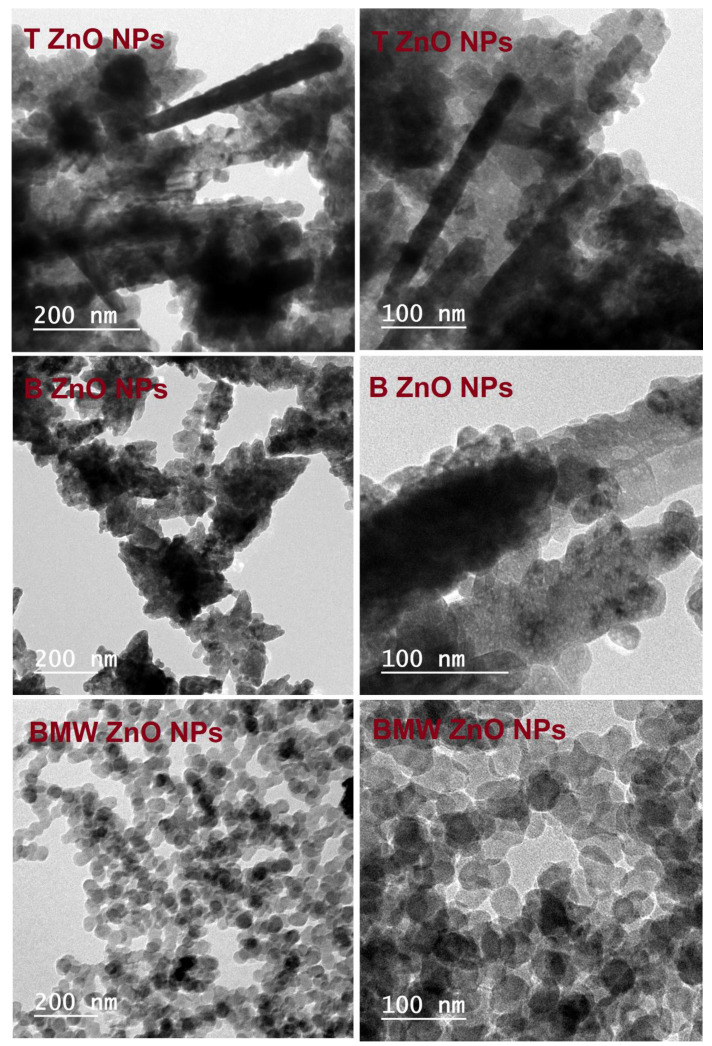
TEM micrographs of different ZnO NPs.

Figure 2 illustrates the XRD patterns of different prepared ZnO NPs; T ZnO NPs, B ZnO NPs and BMW ZnO NPs show characteristic peaks at 2θ angles equals 31.74°, 34.40° and 36.22° corresponding to (100), (002) and (101) planes, respectively, relative to hexagonal wurtzite structure (JCPDS file no. 36–1451). The ZnO NPs calculated crystallite size applying the Scherer equation were 21.5 nm, 25.7 nm, 26.40 nm, for T ZnO NPs, B ZnO NPs and BMW ZnO NPs, respectively, indicating the increase in the crystallite size due to the thermal treatment via the microwave heating, which was characterized with the rapid, and high temperature compared with the traditional method. The increase in crystallite size with the microwave prepared ZnO NPs could be attributed to thermally promoted crystallite growth [44]. Figure 3 presents the SEM images of the different ZnO nanoparticles at different magnification power. The SEM images revealed the formation of irregular agglomerated nanoparticles when the ZnO NPs prepared traditionally (T ZnO NPs) or biosynthesized using the the Ziziphus jujuba leaves extract only (B ZnO NPs), as well as the prepared by the microwave assisted using the Ziziphus jujuba leaves extract (BMW ZnO NPs) characterized with uniform and less agglomeration nanoparticles, and accordingly, the ZnO NPs were characterized with TEM to confirm the shape and size of the nanoparticles. Transmission electron microscope images of the ZnO NPs; T ZnO NPs, B ZnO NPs and BMW ZnO NPs are presented in Figure 4, which shows that ZnO NPs exists in several sizes and shapes depending on the preparation method. For example, T ZnO NPs composed of irregular agglomerated particles, and the B ZnO NPs characterized with the irregular flake-shape composed of very small particles with an average particle size of 20 nm, whereas BMW ZnO NPs characterized with the well-defined spherical-like shape with an average particle size of 25 nm. The small size of the B ZnO NPs may be attributed to the presence of the Ziziphus jujuba leaves extract which act as capping agent and prevent the agglomeration of the ZnO NPs due to the existence of long chain natural products such as the polyphenols in the extract [41,42,45,46,47,48]. In addition, the well-defined spherical shape of the BMW ZnO NPs compared with the traditional and biosynthesized ZnO NPs may be ascribed to the thermally promoted crystallite growth due to the fusion of the small particles as a result of the high temperature, as well as the effect of the long chain natural products associated with the Ziziphus jujuba leaves extract.

**Figure 5:**
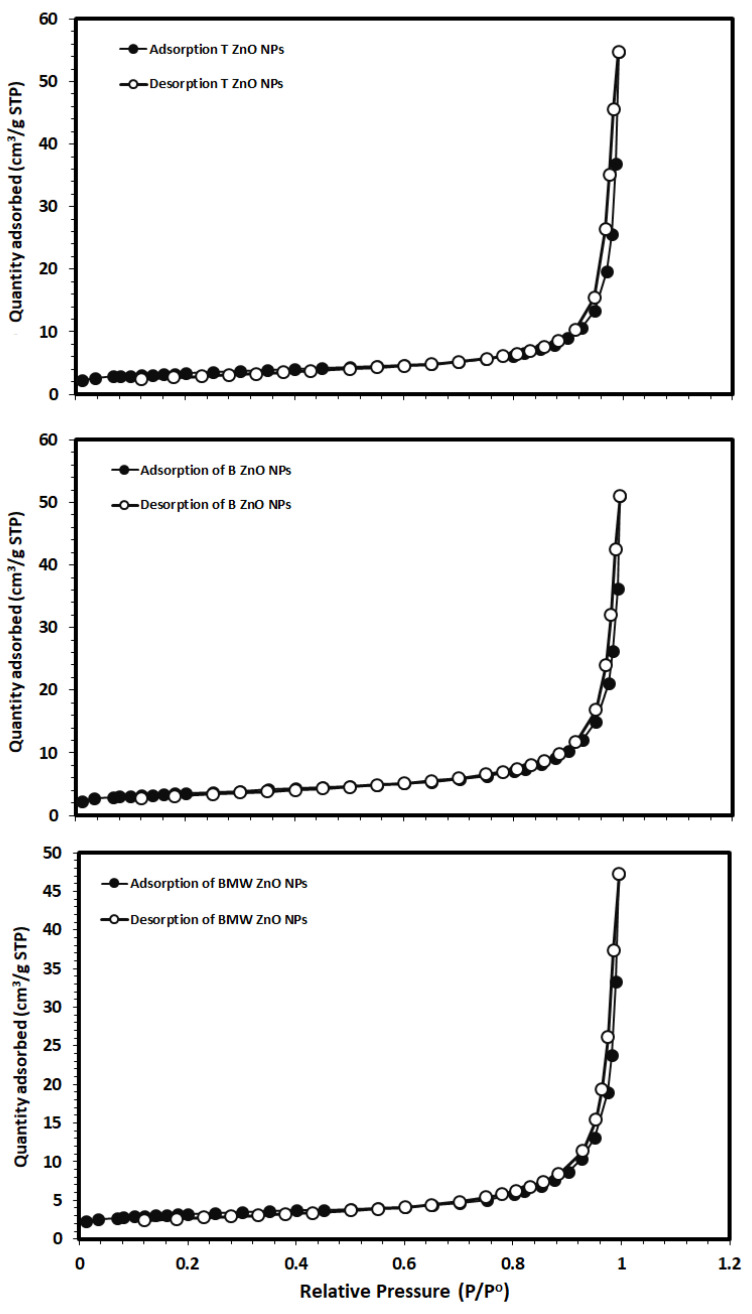
N_2_ adsorption/desorption isotherms of different ZnO NPs.

The specific surface areas of the ZnO NPs were calculated from the nitrogen gas adsorption/desorption isotherms at 77 K applying the BET equation, as shown in Figure 5. The BET specific surface areas were 12.7, 11.5 and 11.4 m^2^/g for the T ZnO NPs, B ZnO NPs and BMW ZnO NPs, respectively, indicating a slight decrease in the surface area upon the microwave treatment. The average particles size (D) of BMW ZnO NPs was estimated based on their spherical shape, calculated specific surface area and the ZnO NPs theoretical density using the following equation [49,50]:

(2) D=(N×1000)/(SSA× ρ)

where D is average particle size of particles (nm), N is the shape coefficient (N= 6 for the spherical shape), SSA is specific surface area m^2^/g and ρ is theoretical density of the ZnO NPs (5.61 g/cm^3^). The calculated average particle size was 98.8 nm, based on the SSA value of 11.4 m^2^/g of the BMW ZnO NPs, which is significantly higher than the TEM value of 25 nm and the crystallite size of crystallite size of 26.40 nm estimated by applying the Scherer equation to the main diffraction peak at 2θ value of 36.22° corresponding to (101) plane.

**Figure 6:**
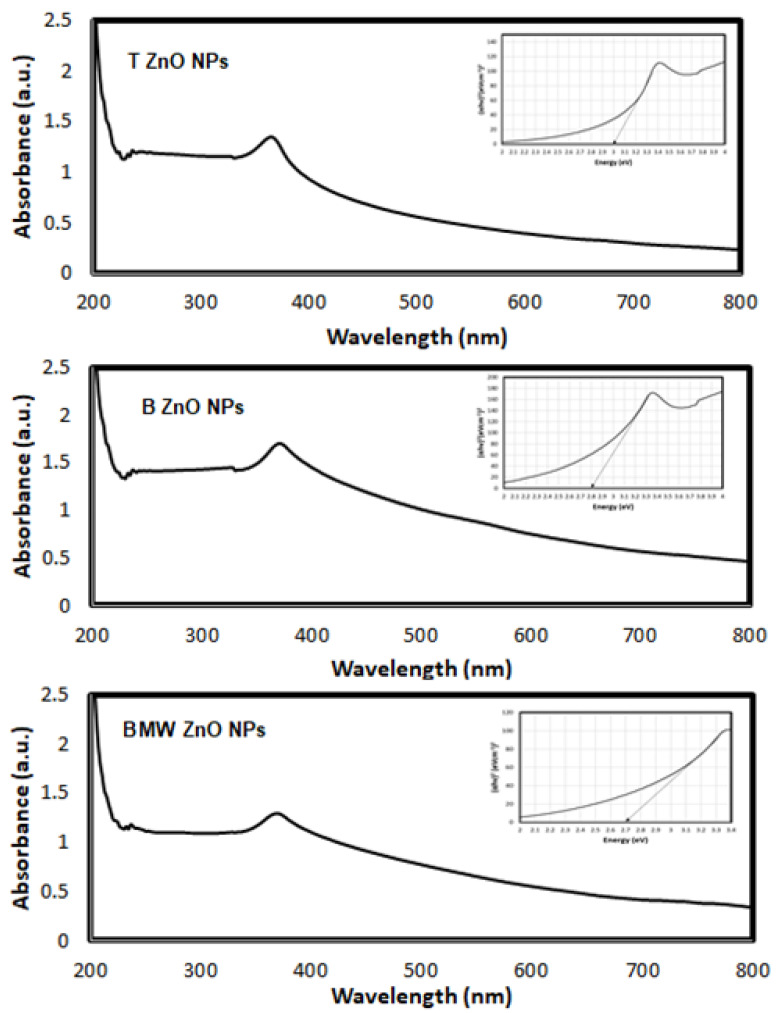
Evaluation of band-gap energy for ZnO photocatalysts.

In order to investigate the optical property of the traditional and biosynthesized ZnONPs on the band gap energy value, the UV–Vis absorbance spectra were recorded at room temperature in the range of 200–800 nm and are shown in Figure 6. As can be seen from figure, the position of the absorption spectra exhibits red shift with the biosynthesis which indicates that the band gap of ZnO material decreases compared with traditional method. It shows the UV–vis absorption spectra of ZnO nanoparticles exhibiting absorption peak at 364 nm, 368 and 370 nm for T ZnO NPs, B ZnO and, BMW, respectively.

The optical band gap energy (Eg) of the samples is determined by fitting the absorption data using Tauc’s relation [51]:

(3) αhυ= E(hυ−Eg)1/2

where hν is the photon energy, Eg is the direct band gap and E is a constant, α is the optical absorption coefficient and found from the absorption data. As presented in Figure 6, plotting (αhν)2 as a function of photon energy (hυ) and extrapolating the linear portion of the curve to zero absorption gives the value of the direct band gap (Eg). It was observed that the band-gap energy was decreased to 2.70, 2.80 and 3.00 eV for BMW ZnO NPs, B ZnO NPs and T ZnO NPs, respectively, which were lower than the typical ZnO (3.2 eV) [52]. Compared to the bulk ZnO, the optical band gap of the prepared ZnO NPs were smaller, which may be due to the structural defects that arising during the sample synthesis. As it is well known, the point effects in ZnO introduce levels within the bandgap lead to the appearance of the wide deep level emission band covering the whole visible range [53]. The wide band gap of ZnO indicates that it is difficult to photo-excite electrons from the valance band (VB) to the conduction band (CB) under visible light. However, the presence of 4 oxygen vacancies in ZnO as surface defects plays an important role in optical absorption in visible light region. Theoretical calculations by first principle density functional theory (DFT) concluded that the valence states near Fermi level originated from the O2p and Zn3d states in the valence band can lead to the electronic transitions in visible region [54,55].

### 3.2. Photocatalytic Degradation of MO and MB 

The photocatalytic degradation of MO and MB was explored using T ZnO NPs, B ZnO NPs and the BMW ZnO NPs under the solar irradiation, and the results revealed that the most of the MO and MB dyes were removed within 100 min using BMW ZnO NPs, 100.0% and 99.6%, respectively, whereas in the case of B ZnO NPs it required 150 min to remove most of the dyes—95.5% and 96.6% removal for MO and MB dyes, respectively, meanwhile in the case of T ZnO NPs it required 180 min to remove most of the dyes—94.2% and 92.6% removal for MO and MB dyes, respectively. The high photodegradation efficacy of the BMW ZnO NPs may be attributed to the small band gap (2.70 eV), compared with the B ZnO NPs (2.80 eV) and T ZnO NPs (3.00 eV), which facilitate the photocatalytic degradation of the organic dyes, as a results of the uniform spherical shape of the biosynthesized microwave treatment of the ZnO NPs, as the geometrical shape play an important role in the reactivity of the nanoparticles [17,56], which presents the unique characteristics of the biosynthesized microwave-assisted ZnO NPs. Accordingly, further photocatalytic degradation experiments were performed using the MWG ZnO NPs. 

**Figure 7:**
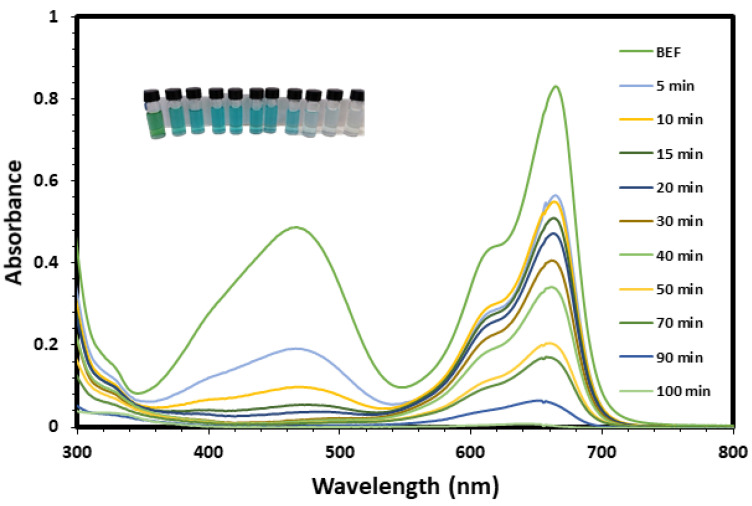
UV-Vis spectra of MO and MB dyes as a function of time in the presence of BMW ZnO NPs.

**Figure 8:**
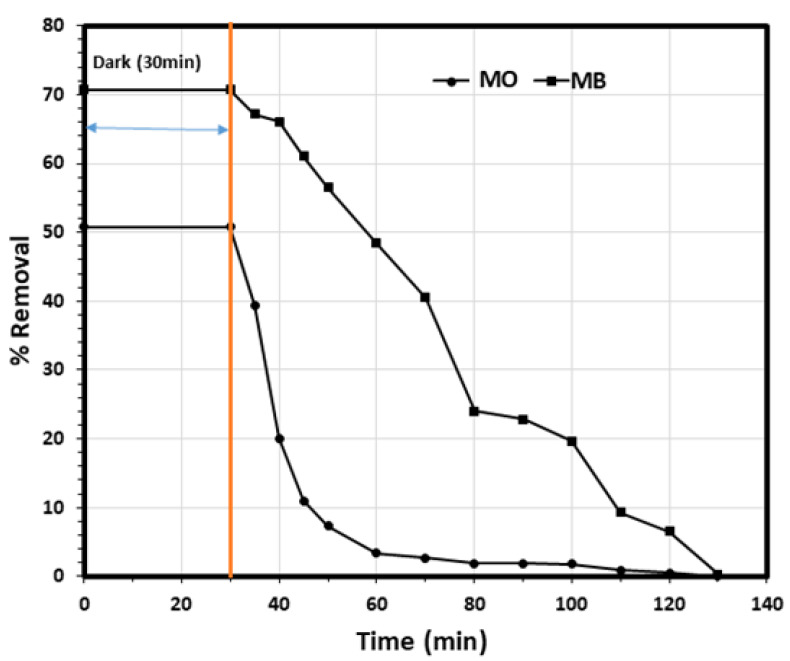
Removal efficiency of a model mixture solution containing MO and MB over BMW ZnO NPs. (Experimental conditions: 20.0 mL solution, pH 6, BMW ZnO NPs mass 15.0 mg, 298 K and [MO] 5.0 mg/L, [MB] 5.0 mg/L).

The environmental application of the biosynthesized ZnO NPs for the removal of MO and MB dyes from aqueous solution was explored and the results were presented in Figure 7 and Figure 8. As it is presented, the first part of the experiment was carried out in the dark for 30 min to study the removal of the pollutants via the adsorption pathway, and the results revealed that the removal efficiencies due to adsorption were 52.3% and 27.2% for the MO and MB, respectively. Then, the photocatalytic properties of ZnO NPs on the degradation of MO and MB dyes were studied under direct sunlight for 100 min, and it was observed that the degradation was generally in two stages. The first stage of the photodegradation was fast, followed by a slower stage. The slow degradation in the second stage might may be due to difficulty to oxidize of the N-atoms of the dye in addition to the accumulated intermediates in the first stage decrease the rate of oxidative photocatalytic reaction [57].

**Figure 9:**
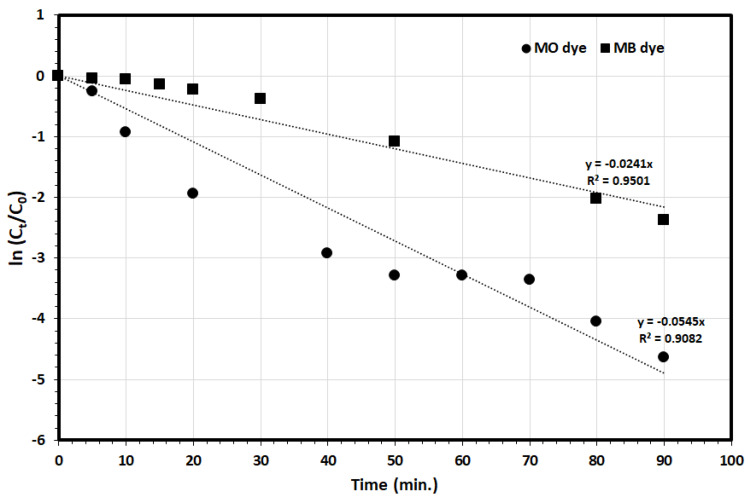
Kinetic plots for the photocatalytic degradation of MO and MB over BMW ZnO NPs. (Experimental conditions: 20.0 mL solution, pH 6, BMW ZnO NPs mass 15.0 mg, 298 K and [MO] 5.0 mg/L, [MB] 5.0 mg/L).

The kinetics of MO, MB dyes photodegradation was explored, using the first order model to determine the kinetics rate in the photodegradation process of MO and MB dyes by the biosynthesized ZnO NPs according to the following equation:

(4) $$\ln\left(\frac{C_{t}}{C_{0}}\right)\;=\;-k\;t$$

where *C*_0_, *C_t_* and *k* are the initial concentration, the concentration at different irradiation times (mg/L) of MO and MB dye, and the photodegradation rate constant (min^−1^), respectively. As it is presented in Figure 9, the plot of ln(*C_t_*/*C*_0_) versus time (t) for both MO and MB dyes photodegradation experimental data showed in Figure 8, yield straight lines with slopes equal to *k* with the linear correlation coefficient value (R^2^) higher than 0.90 indicating that the photodegradation of the MO and MB dyes by the ZnO NPs follows the first order kinetics behavior, with a photodegradation rate constants of 0.0545 min^−1^ and 0.0241 min^−1^ for the MO and MB, respectively, indicating the high photodegradation rate of the MO dye compared with the MB dye by the ZnO NPs. Intriguingly, this may indicate the significant role of the organic dye molecule charge on the removal process as MO is anionic dye whereas MB is a cationic dye. As it was observed earlier, the adsorption (in the dark) of the MO dye molecules was much more compared with the MB dye molecule—52.3% and 27.2%, respectively, indicating the selective nature of the ZnO NPs for the adsorption and further photocatalytic degradation of the MO dye compared with the MB dye.

### 3.3. Photodegradation Mechanism

The photocatalytic degradation reaction of the MO and MB dyes by ZnO NPs generally includes the following steps: photoexcitation, charge separation and migration and finally surface oxidation–reduction reactions [58], as it explained by Equations (5)–(11), the reactive species generated during irradiation of the ZnO NPs were $h_{\left(\mathrm{VB}\right)}^{+}$, ${\mathrm{OH}}^{.}$ and $O_{2}^{-.}$.

(5) $$\mathrm{ZnO}\;\to \mathrm{ZnO}\;\left(e_{\left(\mathrm{CB}\right)}^{-}\right)+\left(h_{\left(\mathrm{VB}\right)}^{+}\right)$$

(6) $$\mathrm{ZnO}\left(h_{\left(\mathrm{VB}\right)}^{+}\right)+{\;H}_{2}O\;\to \mathrm{ZnO}+{\;H}^{+}+\mathrm{OH}$$

(7) $$Z\mathrm{nO}\left(e_{\left(\mathrm{CB}\right)}^{-}\right)+O_{2}\;\to \mathrm{ZnO}+O_{2}^{-.}$$

(8) $$O_{2}^{-.}+H^{+}\to HO_{2}^{.}$$

(9) $$HO_{2}^{.}+HO_{2}^{.}\;\to \;H_{2}O_{2\;}+O_{2}$$

(10) $$H_{2}O_{2\;}\overset{hv}{\to }\;2{\mathrm{OH}}^{.}$$

(11) $$Organic\;pollutants+{\;\mathrm{OH}}^{.}\;\to degradation\;products$$

**Figure 10:**
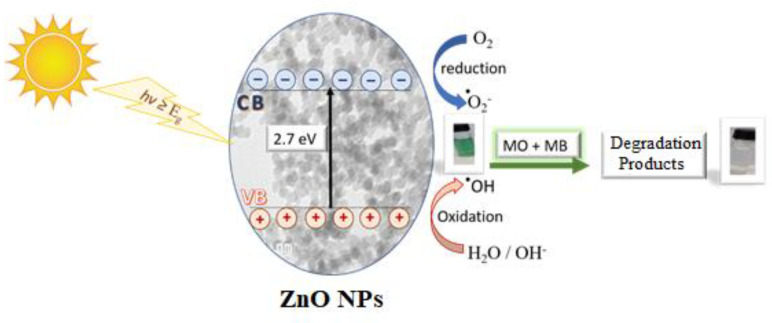
The proposed mechanism for the photo-degradation of MO,MB dyes by the BMW ZnO NPs.

Figure 10 presents the proposed mechanism for photocatalytic degradation of MO and MB dyes by ZnO NPs photocatalyst under solar irradiation. It is proposed that the electrons in the VB transfer to the CB under the solar irradiation of the of the ZnO NPs, and the corresponding energy is higher than the band gap of ZnO (2.7 eV), in that way promoting the generation of valance band holes (h^+^) and conduction band electrons (e^−^), and possibly, the photogenerated holes at the VB could either directly oxidize the adsorbed MO and MB dyes or directly react with hydroxyl (OH^−^) or H_2_O to generate hydroxyl radicals (·OH). Meanwhile, the photoelectrons at the CB could reduce the oxygen (O_2)_ adsorbed on the ZnO NPs surface into superoxide radical (·O_2_^−^). Accordingly, the MO and MB dyes could be decomposed photocatalytically by the both generated ·OH and ·O_2_^−^ [59,60,61].

## 4. Conclusions

Zinc oxide nanoparticles were biosynthesized using *Ziziphus jujuba* leaves extract assisted with microwave and used for the photocatalytic degradation of the methyl orange anionic dye and the methylene blue cationic dye under solar irradiation. The biosynthesized ZnO NPs were characterized and the results showed that ZnO NPs contained hexagonal wurtzite and was characterized with a well-defined spherical-like shape with an average particle size of 25 nm with an outstanding band gap of 2.7 eV and surface area of 11.4 m^2^/g. The photocatalytic degradation of the MO and MB dyes by biosynthesized ZnO NPs under solar irradiation was studied and the results revealed the selective nature of the ZnO NPs for the adsorption and further photocatalytic degradation of the MO dye compared with the MB dye. In addition, the photocatalytic degradation of MO and MB dyes by the ZnO NPs under solar radiation was fitted by the first-order kinetics. Moreover, the photodegradation mechanism proposed that superoxide ions and hydroxyl radicals are the main reactive species.

## Data Availability

Not applicable.
